# Patients With Chronic Hepatitis C Virus Infection Are at an Increased Risk of Colorectal Cancer: A Nationwide Population-Based Case-Control Study in Taiwan

**DOI:** 10.3389/fonc.2020.561420

**Published:** 2021-01-08

**Authors:** Fu-Hsiung Su, Chyi-Huey Bai, Thi Nga Le, Chih-Hsin Muo, Shih-Ni Chang, Arlene Te, Fung-Chang Sung, Chih-Ching Yeh

**Affiliations:** ^1^ Department of Family Medicine, Cardinal Tien Hospital, Fu Jen Catholic University, New Taipei City, Taiwan; ^2^ School of Medicine, College of Medicine, Fu Jen Catholic University, New Taipei City, Taiwan; ^3^ School of Public Health, College of Public Health, Taipei Medical University, Taipei, Taiwan; ^4^ International Master/PhD Program, College of Medicine, Taipei Medical University, Taipei City, Taiwan; ^5^ Management Office for Health Data, China Medical University Hospital, Taichung, Taiwan; ^6^ Big Data Center, China Medical University Hospital, Taichung, Taiwan; ^7^ The Ph.D. Program for Cancer Biology and Drug Discovery, School of Medicine, China Medical University, Taichung, Taiwan; ^8^ Department of Health Services Administration, College of Public Health, China Medical University, Taichung, Taiwan; ^9^ Department of Food Nutrition and Health Biotechnology, Asia University, Taichung, Taiwan; ^10^ Department of Public Health, College of Public Health, China Medical University, Taichung, Taiwan; ^11^ Cancer Center, Wan Fang Hospital, Taipei Medical University, Taipei, Taiwan; ^12^ Master Program in Applied Molecular Epidemiology, College of Public Health, Taipei Medical University, Taipei, Taiwan

**Keywords:** hepatitis C virus, colorectal cancer, population-based, case-control study, insurance data

## Abstract

**Aim:**

Studies evaluating colorectal cancer (CRC) risk associated with chronic hepatitis C virus (HCV) infection are limited.

**Methods:**

In this case-control study, we identify 67,670 CRC cases newly diagnosed from 2005 to 2011 and randomly selected 67,670 controls without HCV and CRC from the same database, frequency matched by age and sex of cases.

**Results:**

Results of logistic regression analysis revealed that the adjusted odds ratio (aOR) of CRC was 1.16 (95% confidence interval [CI] = 1.08–1.24, p < 0.001) in association with chronic HCV. The CRC risk was slightly greater for women than for men. The risk decreased with age, with the aOR decreased from 2.26 (95% CI = 1.32–3.87, p = 0.003) in patients under 45 years old to 1.31 (95% CI = 1.10–1.55, p = 0.03) in patients aged 50–59, and 1.10 (95% CI = 1.00–1.22, p = 0.061) in patients aged over 70.

**Conclusions:**

Our findings suggested that patients with chronic HCV infection are at an elevated risk of developing CRC. Our data also imply that the CRC prevention programs are needed to target younger HCV patients.

## Introduction

Near 20% of worldwide malignancy strain can be associated to various virulent bodies. Hepatitis B virus (HBV) and/or hepatitis C virus (HCV), Epstein Barr virus, human papilloma virus, and *Helicobacter pylori* may contribute to 1,200,000 annual cancer incident cases globally (1). HBV and/or HCV patients are at higher odds of developing liver tumor. However, cancer development requires not only oncoviruses but also several years of continuous infection accompanying chronic inflammation or immune-mediated suppression (2).

The prevalence of anti-HCV ranges from 1.7 to 2.8% in adults worldwide (3, 4). More than 184 million of people in the world have been diagnosed with the chronic infection of HCV in 2005. Both HBV and HCV are the most important causes of hepatocellular carcinoma (HCC) worldwide (4).

HCV infection is prevalent in Asian and African populations, may range from 4.4 to 15% (5, 6). With 4.4% of people aged ≥20 years living with HCV, Taiwan is one of areas with a high HCV infection rate, which increases with age (5).

An inadequate cultural desire of intravenous injections for common cold or fatigue and other minor conditions, and reusing syringes without adequate sterilization were the common causes of HCV infection in Taiwan, as disposable needles and syringes were not commonly available before 1980. And in earlier, unlicensed health care personnel might provide medical care in rural areas (5, 6). An earlier genotype study in hyperendemic areas in Taiwan found that genotype 1b was the most prevalent (47.0 to 76.9%), followed by genotypes 2a and 2b, in 1,164 patients positive for serum HCV antibodies and HCV RNA (ribonucleic acid). The genotype 1b HCV was more prevalent in older age groups, whereas the genotype 2a was more prevalent in younger people (7). A recent study in a southern Taiwan hospital found 18.3% of serum samples tested were genotype 6 among 1,147 patients with hepatitis C viremia (8).

In addition to being a vital risk factor for HCC, HCV infection has been commonly associated with other manifestations. The infection has also been associated with developing type 2 diabetes, lymphoma, neurological disorders, and even extrahepatic cancers and intrahepatic cholangiocarcinoma (9, 10). The extrahepatic malignancy progression in patients with HCV infection is not fully understood, such as the development of colorectal cancer (CRC) (10).

CRC is a highly prevalent cancer in the world and has become the third leading deaths among cancers (11). The CRC incidence rate has drastically increased in the recent decades in Oriental countries, namely Korea, Japan, and China (12). In Taiwan, there was a 30% of increase in CRC incidence rate during 2000–2016, with the second highest incidence and mortality among cancers in 2016 (13). Age, hereditary factors, lifestyle determinants (such as sedentary lifestyle, obesity, red meat consumption, smoking, and alcohol consumption), and long-term bowel inflammation have been associated with CRC in etiologic studies (14). Using colonoscopy to screen 233 participants with chronic HCV infection and 466 controls without HCV infection, the US study found that individuals with chronic HCV were at a 2-fold higher risk of colorectal adenoma in the distal colon than did those without HCV (15). However, limited studies have revealed a discrepancy in the association between CRC and HCV infection (16–20). People in Taiwan have been prevalent with both CRC and HCV infection. We therefore took the advantage of using a large nationwide population-based insurance claims data available to evaluate whether patients with chronic HCV infection are at an increased risk to develop CRC.

## Materials and Methods

### Data Source

This population-based case-control study is conducted by using data obtained from the National Health Insurance (NHI) Research Database (NHIRD) of Taiwan. The NHIRD data were claims data medical providers submitted to the nationalized insurance program that started from 1995 to offer affordable, good-quality, and extensive health care services to Taiwanese residents (21). The insurance program covers nearly 99% of the Taiwanese citizens (22). Detailed information of the program is available in our previous studies (23, 24). This research was approved by the Institutional Review Board of China Medical University and Hospital Research Ethics Committee (Institutional Review Board approval number: CMU-REC-101-012).

### Study Population

The International Classification of Diseases, Tenth Revision (ICD-10) has not been nationally implemented until January 2016, we used International Classification of Diseases, Ninth Revision, Clinical Modification (ICD-9-CM) to identify diseases in the claims data. Patients with a diagnosis of CRC (153–154) from 2005 to 2011 were acknowledged from the Registry for Catastrophic Illness Patient (RCIP) database. The RCIP, an expansion program of the NHI program of Taiwan, was designed for look after people with serious diseases from financial crisis. The NHI program pays for expenses generated for the treatment of the disease when the patient with intractable disease is eligible to register in the RCIP (25). CRC is a NHI-recognized catastrophic illness. For patients with newly diagnosed CRC to be eligible for a catastrophic illness certificate, it is mandatory for the NHI administration to approve after reviewing imaging, clinical, and laboratory information provided by the primary care physician.

The present research evaluated the relationship between CRC risk and chronic HCV infection (coded as ICD-9-CM 070.41, 070.44, 070.51, 070.54, and V02.62). Patients with human immunodeficiency virus (HIV) infection (ICD-9-CM 042, 043, 044, and V08) or HBV infection (coded as ICD-9-CM 070.2, 070.3, and V02.61) were excluded. Anti-HCV antibody and hepatitis B surface antigen were characteristic plasma markers for HCV and HBV infection, correspondingly. Furthermore, data that lacked sex- and age-related information were excluded. Finally, we recruited 67,670 subjects with CRC after excluding three patients infected with HIV, 4,111 patients of HBV carriers, and 64 subjects with lost information on age and sex.

**Figure 1:**
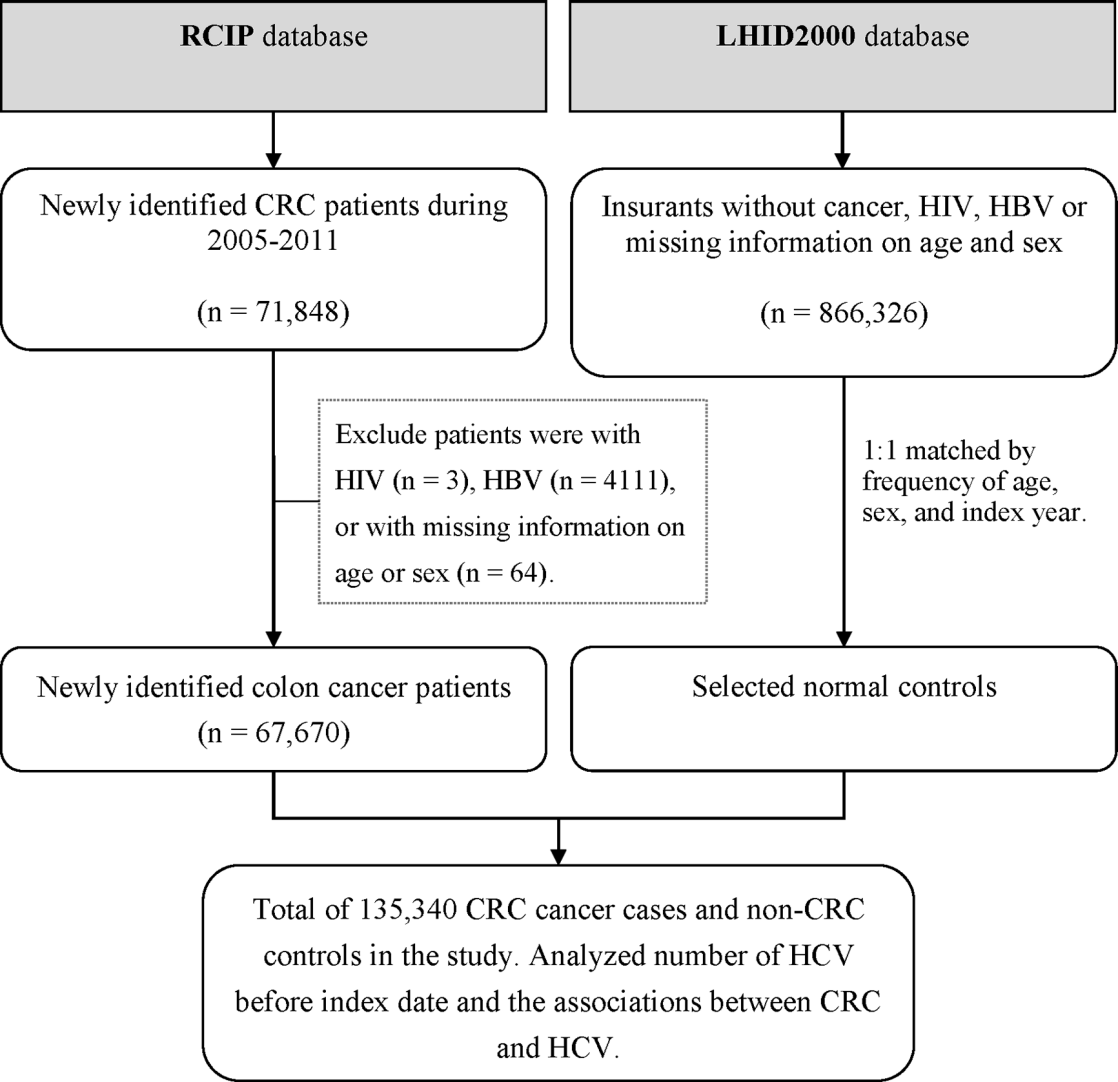
Flow chart for study patients. Patients having colorectal cancer (CRC) were selected from the Registry for Catastrophic Illness Patient (RCIP) database and controls were selected from the Longitudinal Health Insurance Database 2000 (LHID2000) in Taiwan.

Controls were identified randomly from 1 million general population that randomly selected from the NHIRD with claims data between 2000 and 2011. The controls were selected at random from insured population without the history of HIV, HBV, or CRC or with missing data during 2005–2011, frequently matched by age and sex. The age of individual patient was defined as the difference between the index date and the date of birth. Among 866,326 eligible controls, 67,670 were selected. **Figure 1** shows the flowchart for selecting CRC cases and controls from the RCIP database and the 1-million database.

### Statistical Analysis

The distribution of baseline demographic characteristics and comorbidities were compared between CRC cases and controls and examined with Chi-square test. In addition to liver cirrhosis, comorbidities considered as associated covariates included cardiovascular disorders of coronary artery disease (CAD), hyperlipidemia and hypertension, and renal disease, diabetes mellitus (DM), chronic obstructive pulmonary disease (COPD), and obesity ascertained during 2005–2011.

We used logistic regression analysis to calculate odds ratios (ORs) of CRC and 95% confidence intervals (CIs) associated with HCV. The overall relationship was evaluated first and the adjusted OR (aOR) was estimated after controlling for significant covariates using the multivariable analysis. Potential covariates included age, sex, geographical region, occupation, urbanization level, monthly income, DM, hypertension, hyperlipidemia, CAD, renal disease, COPD, obesity, and liver cirrhosis. In addition, we used stratification to differentiate the risk of CRC by covariates. Interactions between chronic HCV infection and covariates on colorectal cancer risk were evaluated using the likelihood ratio test. The 95% CI was used to define the significance of the relationship. Data analyses were performed by using SAS statistical software (version 9.4 for Windows; SAS Institute, Inc., Cary, N.C., USA).

## Results

### Demographic Characteristics

**Table 1** compared the demographic characteristics between CRC cases (N = 67,670) and controls (N = 67,670) identified from 2005 to 2011, matched by age and sex. Statistically significant differences were observed in geographical regions and urbanization levels (p < 0.001), occupation categories (p < 0.001), and monthly incomes (p = 0.020) between the two groups. Hypertension, liver cirrhosis, COPD, CAD, and DM were more prevalent among patients with CRC than among controls (p ≤ 0.001).

**Table 1:** Demographic characteristics and comorbidities compared between colorectal cancer cases and controls during 2005–2011.

Variables	Controls (N = 67,670)		Cases (N = 67,670)		*P* value^*^
	n	(%)	n	(%)	
Sex					1.000
Women	29,208	(43.2)	29,208	(43.2)	
Men	384,62	(56.8)	38,462	(56.8)	
Age, years					1.000
<20	37	(0.05)	37	(0.05)	
20–29	466	(0.69)	466	(0.69)	
30–39	2,079	(3.07)	2,079	(3.07)	
40–49	5,867	(8.67)	5,867	(8.67)	
50–59	13,841	(20.5)	13,841	(20.5)	
60–69	16,035	(23.7)	16,035	(23.7)	
70–79	18,230	(26.9)	18,230	(26.9)	
≥80	11,115	(16.4)	11,115	(16.4)	
Geographical region					<0.001
Northern	28,606	(42.3)	28,726	(42.5)	
Central	13,717	(20.3)	13,512	(20.0)	
Southern	21,413	(31.6)	22,139	(32.7)	
Eastern and islands	3,934	(5.81)	3,293	(4.87)	
Occupation					<0.001
White collar	30,932	(45.7)	32,389	(47.9)	
Blue collar	28,631	(42.3)	28,016	(41.4)	
Retired and others	8,107	(12.0)	7,265	(10.7)	
Urbanization level					<0.001
Urban	18,155	(26.8)	18,669	(27.6)	
Suburban	30,176	(44.6)	30,880	(45.6)	
Rural	19,339	(28.6)	18,121	(26.8)	
Monthly income, NT$					0.020
<15,840	21,316	(31.5)	21,474	(31.7)	
15,841–25,000	32,534	(48.1)	32,054	(47.4)	
≥25,001	13,820	(20.4)	14,142	(20.9)	
Comorbidities					
DM	16,333	(24.1)	18,817	(27.8)	<0.001
Hypertension	36,060	(53.3)	37,593	(55.0)	<0.001
Hyperlipidemia	21,536	(31.8)	21,349	(31.6)	0.275
CAD	19,768	(29.2)	19,236	(28.4)	0.001
Renal disease	10,878	(16.1)	10,883	(16.1)	0.971
COPD	26,535	(39.2)	24,304	(35.9)	<0.001
Obesity	969	(1.43)	1,003	(1.48)	0.441
Liver cirrhosis	15,567	(23.0)	15,047	(22.2)	<0.001

*Chi-square test.

### Colorectal Cancer Risk in Patients With Chronic Hepatitis C Virus Infection

**Figure 2:**
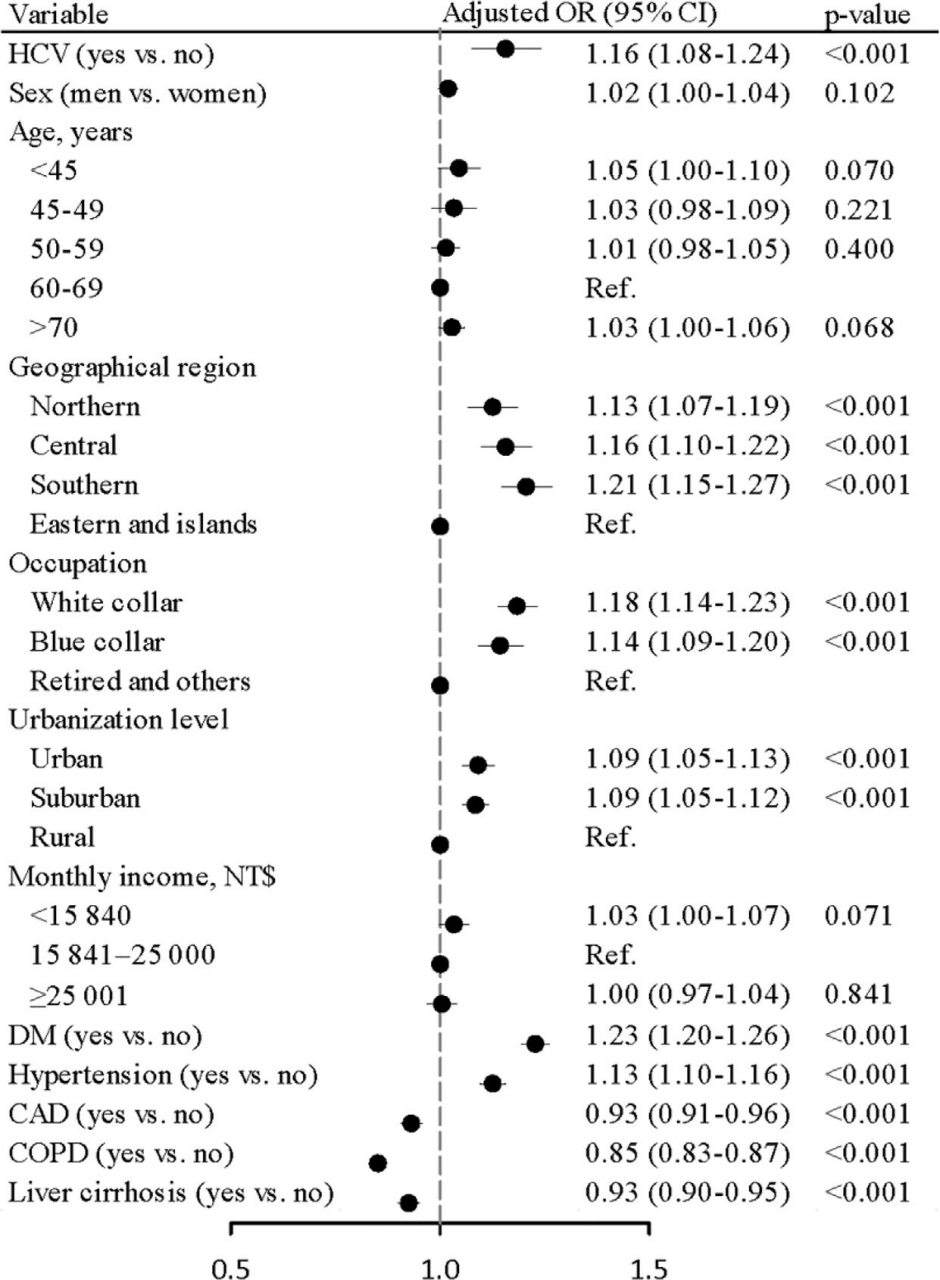
Odds ratios and 95% confidence intervals of colorectal cancer associated with chronic hepatitis C virus infection adjusted for age, sex, geographical region, occupation, urbanization level, monthly income, DM, hypertension, CAD, COPD, and liver cirrhosis.

The prevalence of chronic HCV infection was higher in CRC cases than in controls (2.55 *vs.* 2.30%), with an aOR of 1.16 (95% CI = 1.08–1.24, p < 0.001) after controlling for age, sex, geographical region, occupation, urbanization level, monthly income, DM, hypertension, CAD, COPD, and liver cirrhosis in the multivariable logistic regression model (**Figure 2**). Sensitivity analysis was also performed by subdividing CRC into colon cancer and rectal cancer groups. Results showed that chronic HCV infection was positively linked with the risk of colon cancer (aOR = 1.13, 95% CI = 1.04–1.24, p = 0.007) as well as rectal cancer (aOR = 1.22, 95% CI = 1.08–1.37, p = 0.001) (**Supplementary Table 1**).

### Sex-Specific Colorectal Cancer Risk in Patients With Chronic Hepatitis C Virus Infection

**Figure 3:**
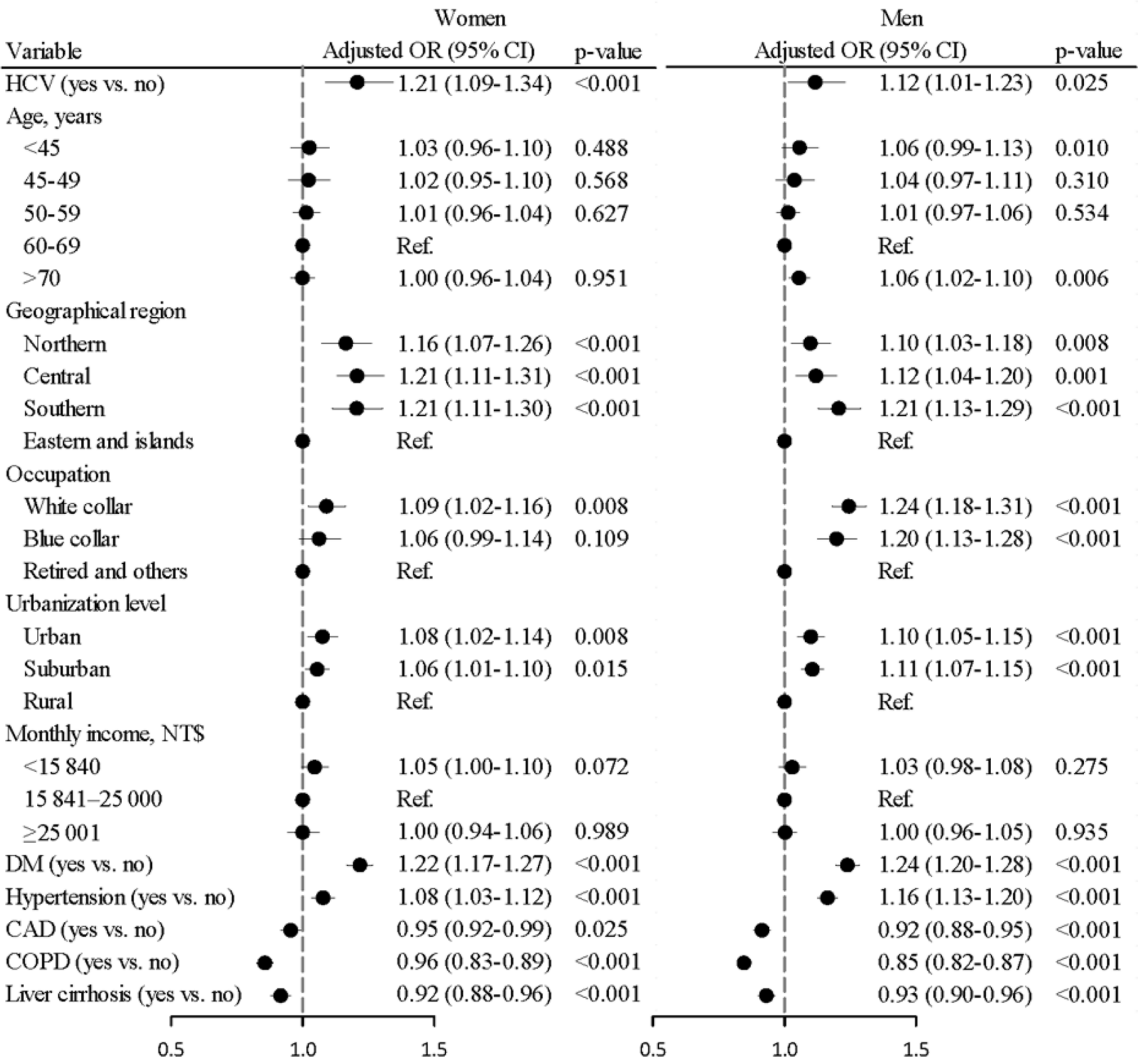
Odds ratios and 95% confidence intervals of colorectal cancer associated with chronic hepatitis C virus infection stratified by sex. (Adjusted for age, geographical region, occupation, urbanization level, monthly income, DM, hypertension, CAD, COPD, and liver cirrhosis) (p for interaction was 0.461).

The HCV infection prevalence was higher in CRC cases than in controls for both females (2.79 *vs.* 2.43%) and males (2.49 *vs.* 2.30%). The sex-specific aORs of CRC associated with HCV infection were 1.21 (95% CI = 1.09–1.34, p < 0.001) in women and 1.12 (95% CI = 1.01–1.23, p = 0.025) in men (**Figure 3**). Further data analysis failed to show a significant interaction between gender and the HCV status on the CRC risk (p = 0.461).

### Age-Specific Colorectal Cancer Risk in Patients With Chronic Hepatitis C Virus Infection

**Figure 4:**
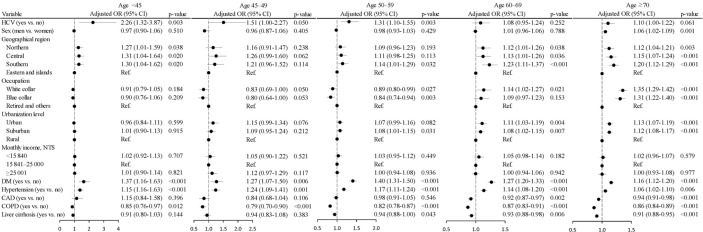
Odds ratios and 95% confidence intervals of colorectal cancer associated with chronic hepatitis C virus infection stratified by age. (Adjusted for sex, geographical region, occupation, urbanization level, monthly income, DM, hypertension, CAD, COPD, and liver cirrhosis) (p for interaction was 0.007).

The aOR of CRC declined from 2.26 (95% CI = 1.32– 3.87, p = 0.003) for HCV patients <45 years old to 1.51 (95% CI = 1.00–2.77, p = 0.050) for HCV patients 45–49 years old, to 1.31 (95% CI = 1.10–1.55, p = 0.003) for HCV patients 50–59 years old, to 1.08 (95% CI = 0.95–1.24, p = 0.252) for HCV patients 60–69 years old, and to 1.10 (95% CI = 1.00–1.22, p = 0.061) for HCV patients ≥70 years old (**Figure 4**). Further data analysis showed a strong interaction between age and the HCV status on CRC risk (p = 0.007). **Supplementary Table 2** showed young HCV patients aged <45 years were at the highest elevated risks of both colon cancer (aOR = 1.99, 95% CI = 1.05–3.77, p = 0.035) and rectal cancer (aOR = 3.27, 95% CI = 1.18–9.03, p = 0.022).

The analyses of CRC risk in patients with chronic HCV infection stratified by geographical region, occupation, urbanization level, monthly income, DM, hypertension, CAD, COPD, and liver cirrhosis were illustrated in the **Supplementary Figures 1**–**9**.

## Discussion

Our population-based case-control study included 67,670 CRC patients and 67,670 controls in an endemic area of chronic HCV infection. Results showed that the HCV infection was higher in CRC cases than in control with an OR of 1.16 (95% CI = 1.08–1.24, p < 0.001). The risk was greater in younger patients. Limited studies have investigated HCV infection in patients with CRC, and the role of HCV infection in CRC development remains unclear. An earlier US study found chronic HCV infection is associated 2-fold higher risk of colorectal adenoma in the distal colon (15). Hurtado-Cordovi and colleagues found an increased incidence of colorectal adenoma (26.3 *vs.* 20.2%) in patients with HCV than controls without HCV, but not significant (26). Moreover, a retrospective chart review study conducted by Prakash et al. showed that patients with HCV had a higher incidence of colorectal adenoma detected from screening colonoscopy than did the general population (27). However, these studies were conducted with small sample sizes, it is difficult to draw a solid conclusion. Another case-control study conducted by Malaguarnera and his colleagues showed a significantly increased prevalence (p < 0.005) of anti-HCV in 66 elderly patients with CRC (35.5%) compared with 300 controls (10.5%) (28). A US retrospective cohort study with 145,210 HCV patients and 13,948,826 individuals without HCV found an increased CRC risk for HCV patients with a relative risk of 1.93 (95% CI = 1.65–2.27) (20). The US Chronic Hepatitis Cohort Study demonstrated that patients with chronic HCV infection had a significantly increased incidence of rectal cancer (standardized rate ratio [SRR] = 2.1; 95% CI = 1.3–2.8) but not colon malignancy (SRR = 0.4; 95% CI = 0.3–0.6). They also found an elevated risk of mortality from rectal cancer (RR = 2.6; 95% CI = 2.5–2.7) (16). Pol et al. suggested that cancer in patients with HCV infection occur frequently at a young age with poor prognosis (10). An Australian cancer registry study suggested that colon and rectal malignancy occurrence rates was not increased in HCV patients (standardized incidence ratio [SIR] = 0.6, 95% CI = 0.5–0.9 and SIR = 0.3, 95% CI = 0.2–0.6, respectively) (17). Likewise, no increased occurrence of rectal and colon cancers was seen in one nationwide, population-based cohort study conducted in Denmark (SIR = 1.0, 95% CI = 0.2–2.9 and SIR = 1.8, 95% CI = 0.4–5.4, respectively) (18). Furthermore, Swart et al. reported no raised occurrence of CRC in HCV patients (SIR = 0.9, 95% CI = 0.4–1.8) (19).

Drug abuse related injection uses are the predominant mode of HCV transmission in Australia, Europe, and United States (29). By contrast, intravenous injections for minor conditions driven by a cultural desire, inadequate sterilization and reuse of syringes, and lack of licensed medical providers were the main causes of HCV infection in Taiwan decades ago (5, 6). Taiwanese people could have thus exposed to HCV decades ago. Cancer may develop after several years (normally after 20–30 years) of continuous infection accompanying chronic inflammation or immune-mediated suppression (2). Hence, the positive association between CRC and chronic HCV infection is evident in Taiwan as a long period of infection promotes the carcinogenic process of the microorganism.

No epidemiological study has analyzed the role of different HCV genotypes in CRC development. HCV subtypes vary among populations. Types 1a and 1b are the most common in population in the United States and Europe (30). In Australia, genotype 1 accounts for 50–55% of HCV cases and genotype 3 accounts for 35–40% (31, 32). The Denmark population are prevalent with genotypes 1a (43%), 3a (39%), and 1b (11%) (33). In Taiwan, the most common genotypes of HCV are 1b, 2a, and 2b. The prevalence rate of genotype 1b is greater in older population (7). In recent years, genotype 6 has been more prevalent in the general Taiwanese population (8). In our study, the odds of HCV infection declined as age increased in both CRC and control groups. Therefore, HCV genotypes associating with developing CRC in Taiwan are unlikely to be subtypes 1a and 1b, which are the most common in the United States and Europe.

The exact mechanism by which chronic HCV infection leads to CRC development remains unknown. Yi and Yuan reviewed several possible mechanisms of HCV-induced hepatocarcinogenesis (34). HCV and its proteins/components trigger oxidative stress and inflammation-signaling cascades and in turn produce reactive oxygen species (ROS). ROS can lead to host genetic mutation and inflammation, consequently causing liver injury. Moreover, HCV disturbs lipid metabolism, which in turns contributes to steatosis. In addition, HCV regulates the cellular proliferation signaling pathway as well as facilitate TGF-β production. Subsequently, TGF-β promotes hepatic stellate cells to secrete excess extracellular matrix, which subsequently leads to liver fibrosis and inflammation causing tumor initiation and progression. Liver damage resulting from the aforementioned cascades may induce repeated hepatocyte generation. Finally, disease progenitors with abnormal proliferation form a “cancer field” and develop into carcinoma (34).

Studies have suggested that HCV and its particles can present in various extrahepatic organs or tissues (35), including intestinal tissues (36). Extrahepatic carcinogenesis of chronic HCV infection is potentially caused by indirect actions of the organism and probably not by the direct viral cytopathic effect (35). Thus, the hepatocarcinogenic mechanisms of chronic HCV contribute partially to CRC formation. Zhang et al. suggested that HCV activates the Ras/Raf/MEK/ERK pathway, resulting in cell proliferation (37). In addition, the core protein, NS5A, of HCV inhibits the tumor suppressor gene p53 and induces the transcription factor NF-κB (38, 39). The over expression of p53 is associated with the formation of advanced, large-sized adenoma, villous histology, and high-grade dysplasia (40). The induction of the NF-κB pathway is correlated with the malignant progression of colon cells (41).

In Taiwan, the Health Promotion Administration has started to subsidize citizens aged 50–69 years for CRC screening every 2 years since January 2004. In 2013, the age range has been expanded to aged 74 years. The screening program for CRC consists of two phases. First, the stool occult blood test is utilized for mass screening. In the second phase, subjects with a positive stool occult blood test are referred for confirmatory colonoscopy. In our study, we found that the risk of CRC decreased with age of patients with chronic HCV infection, with the adjusted OR decreased from 2.26 for aged <45 years to 1.31 for aged 45–49 years and 1.10 for patients aged ≥70 years. We suggested that the screening program for CRC prevention should be considered for younger HCV patients aged in their early 40s (42).

This study has several limitations. First, asymptomatic HCV carriers might not be identified for this study if they had not sought any medical attention. Therefore, in this study, these patients may be misclassified into the control group with no HCV. This misclassification may lead to estimated ORs toward null values and weaken the risk estimation. Second, ICD-10 is a better diagnostic classification system than ICD-9-CM is. However, ICD-10 has not been introduced to the NHI program in Taiwan till January 2016. Using ICD-9-CM codes to identify diseases may have few common coding variants. However, HCV and cancers are well known important disorders to population in Taiwan. Cancers are considered as catastrophic illnesses requiring certificates approval from physicians and the NHI program. The certificate may benefit the patients with lower payments generate by the disease. Hence, the CRC population in Taiwan is represented by these patients. Using ICD-9 might not accurately detect chronic HCV cases. In addition to anti-HCV, HCV RNA needs to be positive. Hence, using ICD-9 cannot provide the status of the patient’s HCV viremia and treatment. However, previous Taiwanese community epidemiological studies showed that more than 70% of Taiwanese adults with serum anti-HCV positivity were positive to HCV RNA (43–46). Prior to year 2011, less than 10 percentages of the estimated 400,000 Taiwanese HCV with positive RNA cases have successfully reached sustained viral response (SVR) after completing their HCV interferon therapy (47). As result, we feel ICD-9 can provide relatively representative status of HCV prevalence in Taiwan. Third, in this study, information on other potential risk factors was unavailable in the database, including diet, lifestyle, obesity, and family history of CRC (14). Therefore, in the data analysis, we controlled for COPD, CAD, hypertension, obesity, liver cirrhosis, and hyperlipidemia as they may be the result of poor lifestyle modification. Fourth, HCV-infected patients might have more clinic visits leading to more detection of colorectal cancer. However, since January 2004, the Taiwanese national cancer screening program initiated by the Health Promotion Administration has started to subsidize citizens aged 50–69 years for colorectal cancer screening every 2 years. The age range was expanded to 50–74 years in June 2013 (42). Hence, detection bias in the association between HCV infection and colorectal cancer can be minimized by this nationwide screening intervention. Fifth, the HCV transmission route in Taiwan might be different from that in other ethnic groups. Hence, the results of this study should be cautiously interpreted before generalizing to other racial/ethnic groups.

## Conclusions

Our study suggests that patients with chronic HCV infection are at significant risk of developing CRC. The CRC risk could be greater for the younger individuals with HCV infection. It is necessary to conduct in other regions or for other ethnic population to clarify the relationship between CRC risk and chronic HCV infection in addition to the underlying pathophysiological mechanisms.

## Data Availability Statement

The data analyzed in this study is subject to the following licenses/restrictions: Information were gathered from the NHIRD of Taiwan and request can be made by sending a formal proposal to the NHI. Requests to access these datasets should be directed to nhird.nhri.org.tw.

## Ethics Statement

The studies involving human participants were reviewed and approved by the Institutional Review Board of China Medical University and Hospital Research Ethics Committee (Institutional Review Board approval number: CMU-REC-101-012). Written informed consent for participation was not required for this study in accordance with the national legislation and the institutional requirements.

## Author Contributions

F-HS, C-CY, and F-CS conceptualized the study. F-HS, C-HB, TL, F-CS, and C-CY contributed to the methodology. F-HS, C-HM, S-NC, F-CS, and C-CY conducted the formal analysis. F-HS, C-HB, TL, C-HM, AT, F-CS, and C-CY conducted the investigation. F-HS, TL, and C-CY wrote the original draft. F-HS, C-HB, AT, F-CS, and C-CY reviewed and edited the manuscript. All authors contributed to the article and approved the submitted version.

## Funding

Current research is funded by the Ministry of Health and Welfare, Taiwan (MOHW109-TDU-B-212-114004 and MOHW109-TDU-B-212-134020), Children’s Hospital of China Medical University (DMR-108-045), China Medical University Hospital (DMR-109-027 and DMR-109-175), Academia Sinica Stroke Biosignature Project (BM10701010021), MOST Clinical Trial Consortium for Stroke (MOST107-2321-B-039-004), Tseng-Lien Lin Foundation, Taichung, Taiwan, and Katsuzo and Kiyo Aoshima Memorial Funds, Japan.

## Conflict of Interest

The authors declare that the research was conducted in the absence of any commercial or financial relationships that could be construed as a potential conflict of interest.

## Abbreviations

aOR, Adjusted OR; CAD, coronary artery disease; CI, confidence interval; COPD, chronic obstructive pulmonary disease; CRC, colorectal cancer; DM, diabetes mellitus; HBV, hepatitis B virus; HCC, hepatocellular carcinoma; HCV, hepatitis C virus; HIV, human immunodeficiency virus; ICD-9-CM, international classification of diseases, ninth revision, clinical modification; LHID, longitudinal health insurance database; NHI, national health insurance; NHIRD, national health insurance research database; NT$, new Taiwan dollar; OR, odds ratio; RCIP, registry for catastrophic illness patient; RNA, ribonucleic acid; ROS, reactive oxygen species; RR, relative risk; SIR, standardized incidence ratio; SRR, standardized rate ratio.
