# Supplementary material for: Patients With Chronic Hepatitis C Virus Infection Are at an Increased Risk of Colorectal Cancer: A Nationwide Population-Based Case-Control Study in Taiwan
Source: Front Oncol. 2021 Jan 8;10:561420. doi: 10.3389/fonc.2020.561420 (PMC7819899; doi:10.3389/fonc.2020.561420)
Supplement: Supplementary file 1 [file DataSheet_1.doc]

**Supplementary Table 1. Risk of colorectal cancer linked with chronic hepatitis C virus infection stratified by tumor growth site.**

|  |  | Controls |  | Cases |  | Crude |  | Adjusted |  |
| --- | --- | --- | --- | --- | --- | --- | --- | --- | --- |
| Site | HCV | n | (%) | n | (%) | OR (95% CI) | *P* value | OR (95% CI)^a^ | *P* value |
| Colon |  |  |  |  |  |  |  |  |  |
|  | No | 40156 | (97.5) | 40074 | (97.3) | 1.00 (ref) |  | 1.00 (ref) |  |
|  | Yes | 1018 | (2.47) | 1100 | (2.67) | 1.08 (0.99-1.18) | 0.071 | 1.13 (1.04-1.24) | 0.007 |
| Rectum |  |  |  |  |  |  |  |  |  |
|  | No | 25957 | (98.0) | 25868 | (97.6) | 1.00 (ref) |  | 1.00 (ref) |  |
|  | Yes | 539 | (2.03) | 628 | (2.37) | 1.17 (1.04-1.31) | 0.009 | 1.22 (1.08-1.37) | 0.001 |

^a^ Adjusted for age (<45, 45-49, 50-59, 60-69, ≥70), sex, geographical region, occupation, urbanization level, monthly income, DM, hypertension, CAD, COPD, and liver cirrhosis.

**Supplementary Table 2.** Age-predominant risk of colorectal cancer linked with chronic hepatitis C virus infection stratified by tumor growth site.

|  |  | Age <45 | |  | Age 45-49 | |  | Age 50–59 | |  | Age 60–69 | |  | Age ≥70 | |  |
| --- | --- | --- | --- | --- | --- | --- | --- | --- | --- | --- | --- | --- | --- | --- | --- | --- |
| Site | HCV | Co/Ca | OR (95% CI)^a^ | *P* value | Co/Ca | OR (95% CI)^a^ | *P* value | Co/Ca | OR (95% CI)^a^ | *P* value | Co/Ca | OR (95% CI)^a^ | *P* value | Co/Ca | OR (95% CI)^a^ | *P* value |
| Colon |  |  |  |  |  |  |  |  |  |  |  |  |  |  |  |  |
|  | No | 2810/  2796 | 1.00 (ref) |  | 2094/  2083 | 1.00 (ref) |  | 8003/  7961 | 1.00 (ref) |  | 9226/  9214 | 1.00 (ref) |  | 18023/  18020 | 1.00 (ref) |  |
|  | Yes | 15/29 | 1.99 (1.05-3.77) | 0.035 | 25/36 | 1.46 (0.86-2.48) | 0.159 | 156/198 | 1.30 (1.04-1.61) | 0.020 | 277/289 | 1.08 (0.91-1.28) | 0.383 | 545/548 | 1.07 (0.94-1.21) | 0.308 |
| Rectum |  |  |  |  |  |  |  |  |  |  |  |  |  |  |  |  |
|  | No | 1886/  1875 | 1.00 (ref) |  | 1598/  1591 | 1.00 (ref) |  | 5590/  5563 | 1.00 (ref) |  | 6365/  6357 | 1.00 (ref) |  | 10518/  10482 | 1.00 (ref) |  |
|  | Yes | 5/16 | 3.27 (1.18-9.03) | 0.022 | 16/23 | 1.64 (0.85-3.17) | 0.140 | 92/119 | 1.32 (0.99-1.75) | 0.055 | 167/175 | 1.12 (0.90-1.40) | 0.326 | 259/295 | 1.18 (0.99-1.40) | 0.064 |

^a^ Adjusted for sex, geographical region, occupation, urbanization level, monthly income, DM, hypertension, CAD, COPD, and liver cirrhosis.

OR: odds ratio; CI, confidence interval; Co/Ca: Controls/Cases.

p for interaction was 0.069 for colon and 0.163 for rectum
