# Supplementary figures and images for: Patients With Chronic Hepatitis C Virus Infection Are at an Increased Risk of Colorectal Cancer: A Nationwide Population-Based Case-Control Study in Taiwan
Source: Front Oncol. 2021 Jan 8;10:561420. doi: 10.3389/fonc.2020.561420 (PMC7819899; doi:10.3389/fonc.2020.561420)

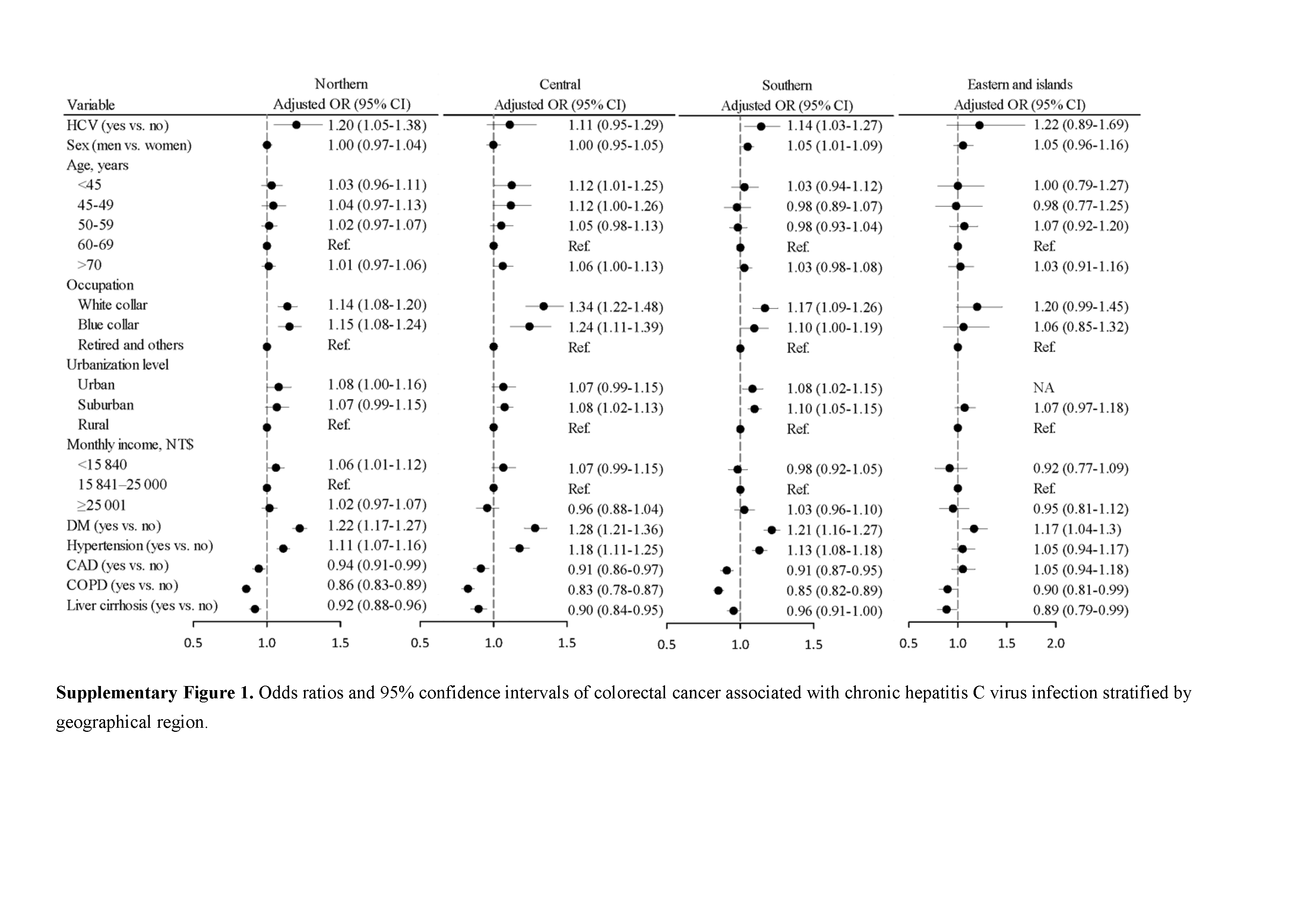

Supplement: Supplementary file 2 [file Image_1.tif]

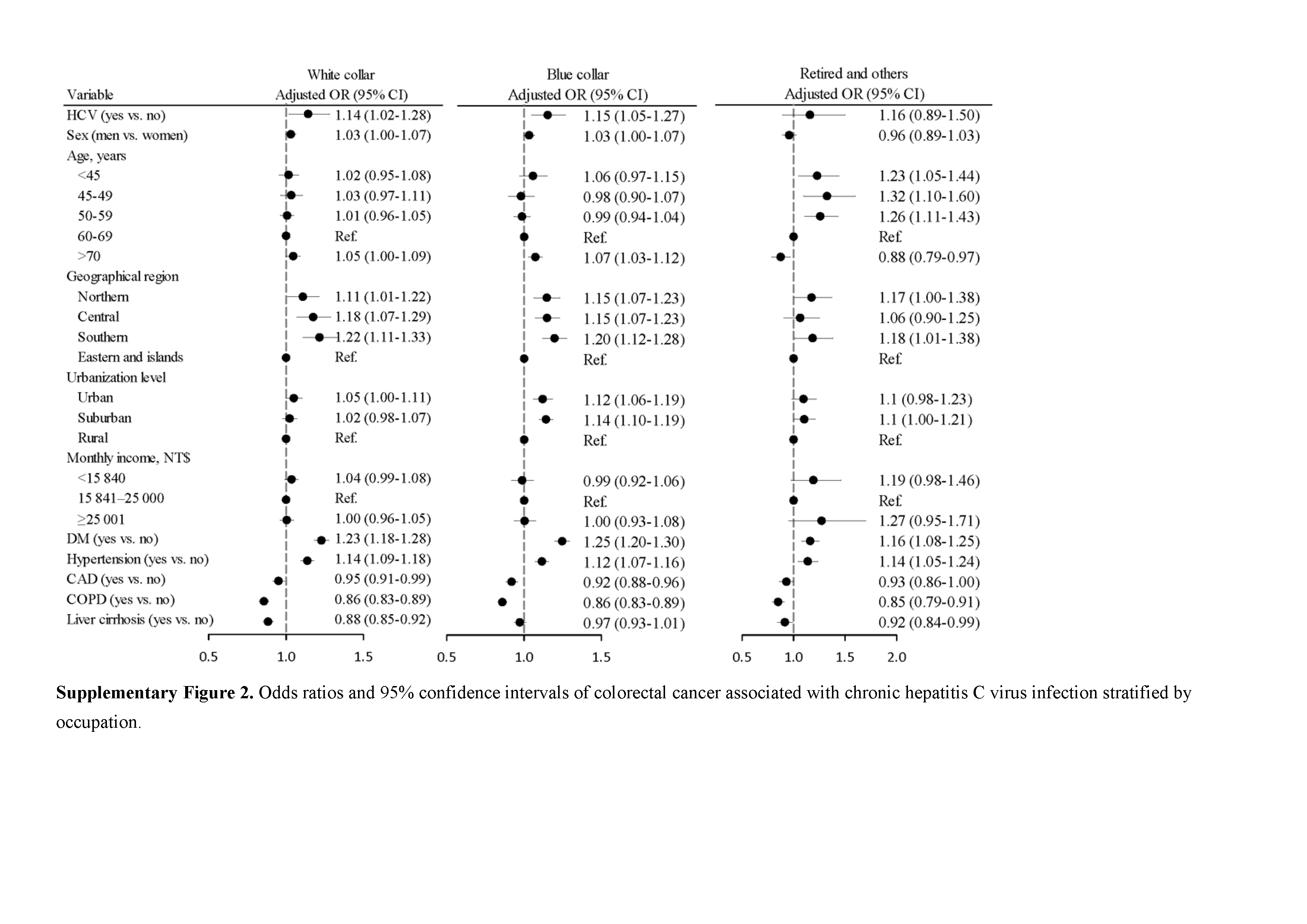

Supplement: Supplementary file 3 [file Image_2.tif]

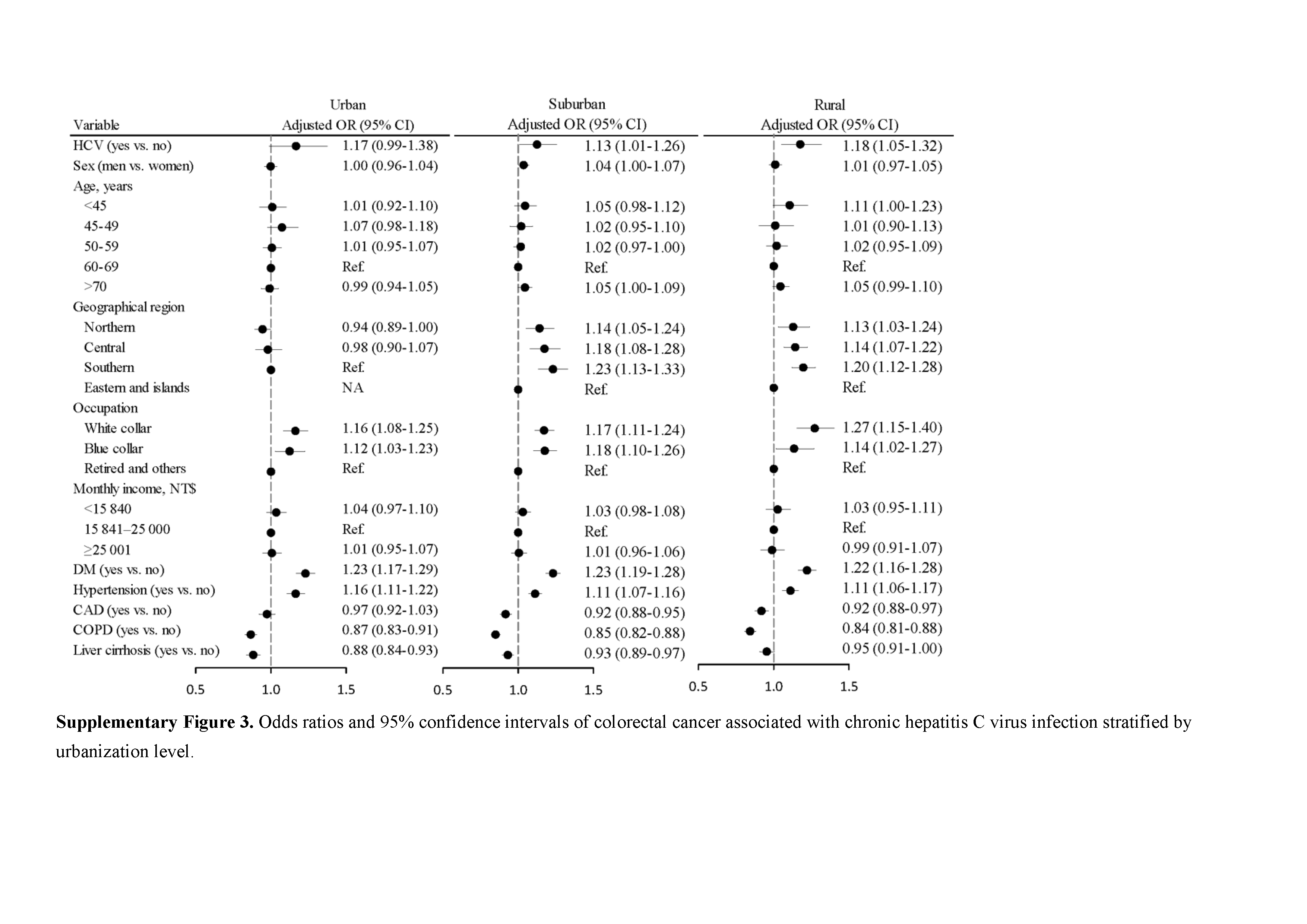

Supplement: Supplementary file 4 [file Image_3.tif]

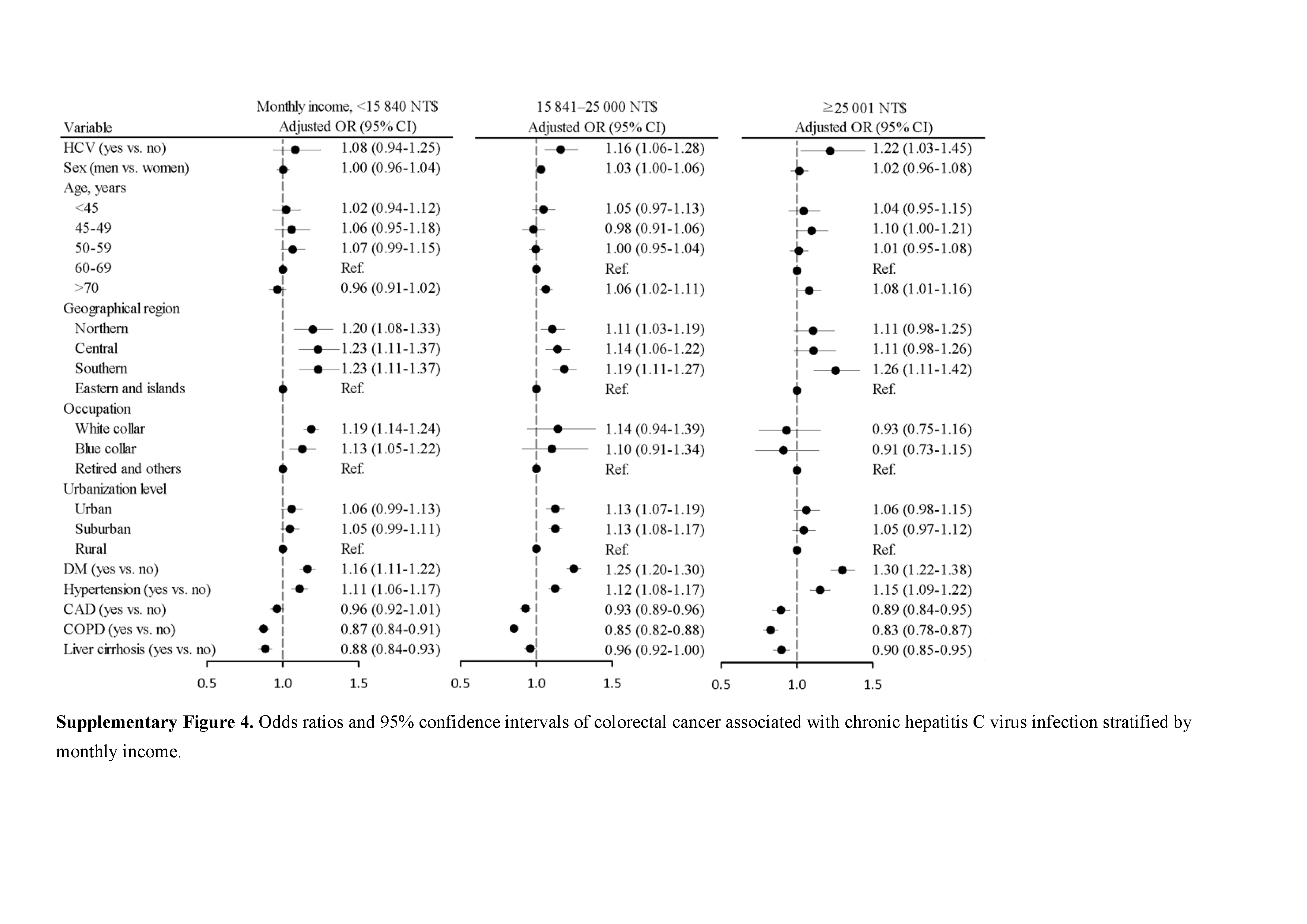

Supplement: Supplementary file 5 [file Image_4.tif]

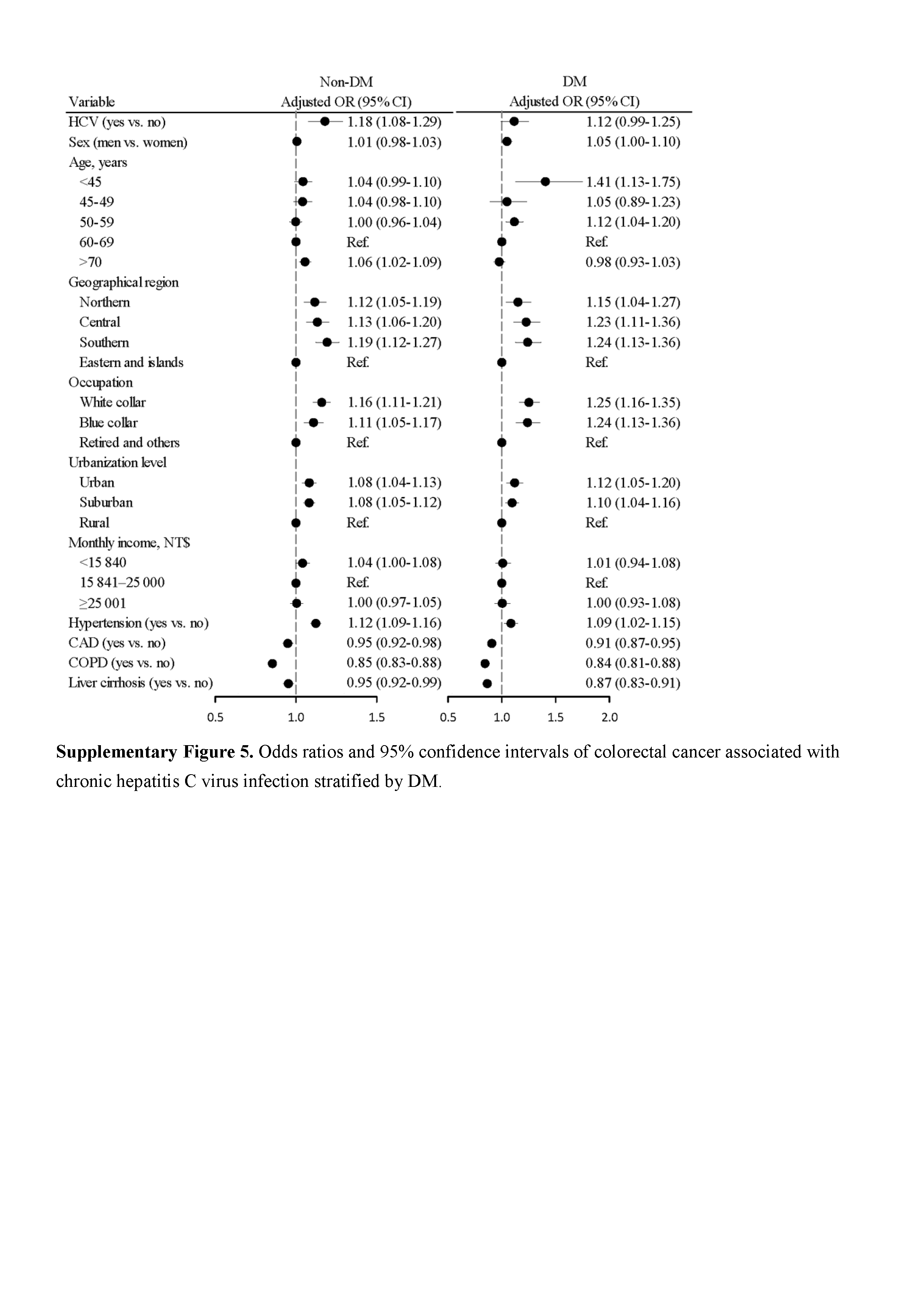

Supplement: Supplementary file 6 [file Image_5.tif]

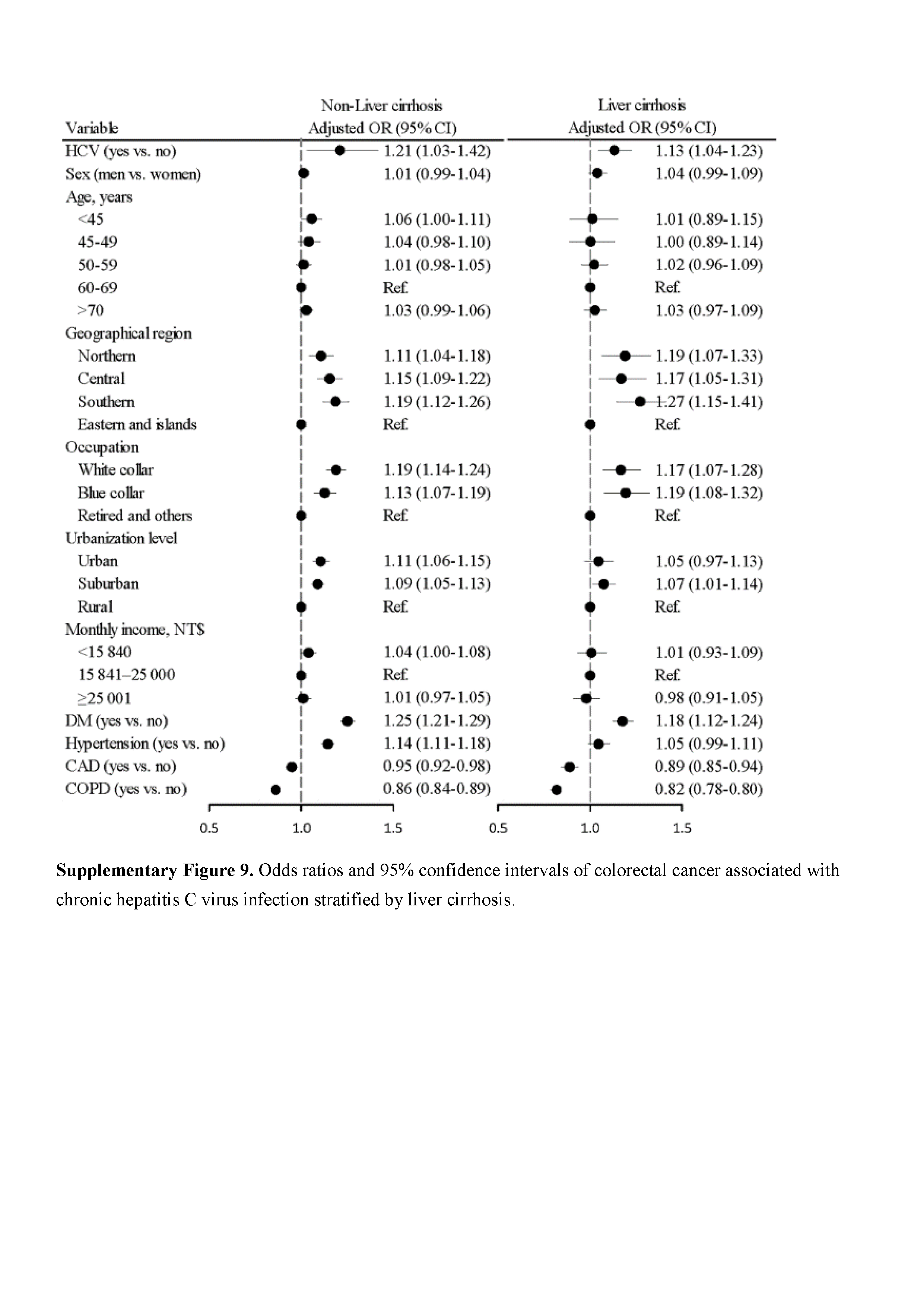

Supplement: Supplementary file 10 [file Image_9.tif]
